# Impact of Polymer Molecular Weight on Aging of Poly(Ethyleneoxide)/Dextran All-Aqueous Emulsions Stabilized by Oppositely Charged Nanoparticle/Polyelectrolyte Assemblies

**DOI:** 10.3390/polym17172305

**Published:** 2025-08-26

**Authors:** Attila Kardos, Mónika Bak, Emese Kovács, György Juhász, Mihály Cserepes, József Tóvári, Róbert Mészáros

**Affiliations:** 1 Institute of Chemistry, Eötvös Loránd University, Pázmány Péter sétány 1/A, H-1117 Budapest, Hungary; 2 Department of Chemistry, Selye J. University, 945 01 Komarno, Slovakia; 3 The National Tumor Biology Laboratory, Department of Experimental Pharmacology, National Institute of Oncology, Ráth György u. 7, H-1122 Budapest, Hungarytovari.jozsef@oncol.hu (J.T.)

**Keywords:** all-aqueous, bijel, emulsion, interfacial assemblies

## Abstract

Aqueous two-phase systems (ATPSs) based on two incompatible polymers have recently garnered considerable attention due to the promising characteristics of all-aqueous emulsions for a range of applications. Recent investigations have indicated strong potential for interfacial assemblies of oppositely charged components in the stabilization of these emulsions. The formation of these confined assemblies is likely to depend on the size of the ATPS-constituting polymers; however, the role of this parameter remains to be elucidated. The primary objective of this study was to examine the effect of polyethylene oxide (PEO) molecular weight on the aging processes of PEO/dextran emulsions that are stabilized by the interfacial association of oppositely charged silica particles and polycations. It has been demonstrated that the stability of emulsions containing one high-molecular-weight dextran is significantly enhanced by increasing the size of the PEO molecules. Furthermore, a compression-induced bijel formation was observed in the ATPS with the largest molecular weight PEO sample. The observations were explained by the impact of the rheology of the aqueous phases on the aggregation, adsorption, and network formation capabilities of polycation/silica assemblies. These findings may facilitate the design of stable all-aqueous emulsions with optimal molecular weights for the ATPS-forming polymers.

## 1. Introduction

Emulsions are well-known colloidal systems that contain liquid droplets dispersed in a continuous liquid phase. They are used in cosmetics and food products [1,2], in multiphasic [3] and catalytic hydrogenation reactions [4], for the synthesis of smart materials or degradable nanocomposites [5,6], and to improve tertiary oil recovery [7,8]. Bicontinuous Pickering emulsion gels (bijels) are a more recent type of structured emulsion [9,10]. The controlled transport of molecules between bicontinuous liquid domains has exciting potential applications in crossflow reactors and biphasic catalysis [10], biomedical tools [11], fibers [12], battery electrolytes [13], and porous systems [14].

One limitation of many earlier emulsion systems is that they contain organic liquid phases. Thus, all-aqueous emulsions have recently received particular attention due to their nontoxic and environmentally friendly nature [15,16]. Aqueous two-phase systems (ATPSs) typically contain two incompatible polymers, one polymer and a salt, or two types of salts or surfactant mixtures. These systems favor the segregation of solutes into different aqueous phases [17,18]. Due to the increasing demand for biocompatible and biodegradable formulations, ATPS mixtures based on biomacromolecules or synthetic polymers, such as poly(ethylene oxide) and dextran, with a water content of up to 80 percent by weight, are currently being targeted [17].

Recent applications of these water-in-water emulsions include encapsulating bacteria [19] and cells [20] and developing bioprinting tools [21]. Furthermore, synthesizing all-aqueous bijels with controlled domain sizes could pave the way for pioneering technologies in biomedical and energy storage applications [10].

Despite their promising features, preparing ATPS emulsions with long-term stability remains challenging due to their low surface tension (γ, roughly between 1 and 0.001 mN/m) and large interface thickness (100–200 nm) [17,22]. The stabilization of all-aqueous emulsions has been achieved using functionalized microgels [23], cellulose nanocrystals [24], terpolymer [25] or biopolymer assemblies [26], and cross-linking of adsorbed Pickering particles [27]. Synthesizing ATPS-based bijels is even more challenging. Previously reported all-aqueous “bijels” have revealed structures with irregular, non-totally interpenetrating liquid domains that may slowly coarsen upon aging [28].

An alternative strategy for stabilizing water/water (W/W) interfaces is forming an interfacial membrane by confining the complexation of oppositely charged polyelectrolytes and/or nanoparticles, which are initially dispersed separately in the adjacent aqueous phases. For example, the interfacial complexation of oppositely charged polyelectrolytes or silica nanoparticles initially separated in polyethylene oxide (PEO) and dextran solutions can result in highly stable emulsion droplets [29,30]. Emulsions with a bicontinuous-like structure have also been observed at specific mass ratios of oppositely charged silica particles [30]. Emulsion stabilization can also be achieved using interfacial assemblies of nanoparticles with oppositely charged macromolecules, including proteins [31], or linear polycations [32].

Recent investigations have clearly demonstrated the significant potential of interfacial membrane formation in stabilizing all-aqueous emulsions. However, the size of the ATPS-forming polymers can significantly affect the transport and association of the oppositely charged components, thus affecting their stabilization efficacy. This phenomenon has yet to be explored. To shed light on these effects, we investigated three PEO/dextran ATPS systems using the same high-molecular-weight polysaccharide and PEO samples with different molecular weights. These systems were stabilized by the interfacial association of negatively charged silica particles and the cationic polyelectrolyte poly(diallyldimethylammonium chloride) (PDADMAC). As we will demonstrate, the PEO molecular weight significantly affects the formation and the adsorption of PDADMAC/silica aggregates, as well as the stability and structure of the resulting emulsions.

## 2. Materials and Methods

### 2.1. Materials

To produce ATPSs, two hydrophilic, incompatible polymers—namely, dextran and poly(ethylene oxide) (PEO)—were utilized. One dextran sample (dextran from *Leuconostoc* spp. M_w_ ≈ 450,000–650,000 Da) and three PEO samples with different average molecular weights—namely, M_n_ ≈ 4000 Da, M_n_ ≈ 20,000 Da, and M_v_ ≈ 100,000 Da—were employed. For the purpose of further designation of the ATPS-constituting polymer samples, dextran, PEO 4 kDa, PEO 20 kDa, and PEO 100 kDa annotations, respectively, will be utilized throughout the present document. As emulsion stabilizers, a 20 wt% aqueous solution of poly(diallyldimethylammonium chloride) (PDADMAC) with M_n_ ≈ 100–200 kDa and a 40 wt% aqueous dispersion of LUDOX^®^ TM-40 colloidal silica nanoparticles were used. Prior to usage, the pH of the silica stock dispersion (pH = 9.7) and the particle diameter (33 nm, as determined by DLS using a Zetasizer Nano ZSP from Malvern Panalytical) were measured. For the purpose of fluorescence imaging, fluorescein isothiocyanate–dextran conjugate (FITC-dextran) was used, with M_w_ ≈ 500 kDa (FITC:Glucose = 1:100). The chemicals utilized in this study were procured from Sigma-Aldrich. The PEO 100 kDa sample was purified via ultracentrifugation (Beckman Coulter Optima XPN-100, Ti-90 rotor) at 50,000 rpm for 4 h at 25 °C to remove insoluble by-products. A 15 wt% PEO stock solution was prepared, and the supernatant obtained after centrifugation was collected and homogenized. The PEO concentration was determined by triplicate dry-mass measurements. All other reagents were used as received. Milli-Q water (TOC < 5 ppb, conductivity > 18 MΩ·cm) was utilized for the preparation of all mixtures and solutions.

### 2.2. Characterization of Phase Behavior

The binodal curves of the three investigated PEO/dextran ATPSs with different molecular weights of PEO samples were determined using turbidimetric titration. For their preparation, stock solutions of 20 wt% for the PEO 4 kDa and 20 kDa samples, 14 wt% for the PEO 100 kDa sample, and 25 wt% for the dextran sample, respectively, were used. PEO/dextran aqueous mixtures of approximately 1 g were prepared with predefined compositions (by weight) within the two-phase region at 25 °C in 4 mL sample vials. The mixtures were subjected to homogenization by means of vortex mixing (IKA Vortex 3) at 2500 rpm for a duration of 60 s. Thereafter, aliquots of Milli-Q water were added in incremental steps, and the total weight of the mixture was documented after each addition. After each dilution step, the samples were subjected to further mixing via the use of a vortex mixer. This process was continued until the mixtures became optically clear, indicating that the composition had reached the single-phase region of the phase diagram. In order to verify the absence of phase boundaries, 1 mL of the samples was centrifuged at 20,000 rpm for 15 min using a Hettich Mikro 200R microcentrifuge. The final compositions of the mixtures were calculated based on cumulative weight measurements.

In order to ascertain compositions with equal volumes of the top and bottom aqueous phases, PEO/dextran ATPSs were meticulously prepared within the two-phase region in Eppendorf tubes with a total volume of 500 μL. For their preparation, stock solutions of 20 wt% for the PEO 4 and 20 kDa samples, 14 wt% for the PEO 100 kDa sample, and 25 wt% for the high-molecular-weight dextran sample, respectively, were used. The mixtures were then homogenized by vortex mixing at 2500 rpm for 60 s and subsequently subjected to centrifugation at 20,000 rpm for 15 min using a Hettich Mikro 200R microcentrifuge. The volume ratios of the two aqueous phases were determined visually for each PEO/dextran system using Eppendorf tubes. Prior to the measurement of the samples, the volumetric scale of the tubes was calibrated by filling them with Milli-Q water in 50 μL increments between 100 and 350 μL.

### 2.3. Emulsion Preparation

Water-in-water emulsions were meticulously prepared in 4 mL sample vials with a total weight of 1.5 g. For the preparation of polymer solutions, stock solutions of 20 wt% for the PEO 4 and PEO 20 kDa samples, 14 wt% for the PEO 100 kDa sample, and 30 wt% for the dextran sample, respectively, were used. In the case of fluorescent emulsions, 5 wt% of the dextran content was FITC-dextran. A silica stock dispersion was prepared by first diluting the 40 wt% commercial solution and subsequently filtering it through a 0.1 μm cellulose acetate syringe filter prior to use. A 2 wt% PDADMAC solution was prepared by first diluting the 20 wt% stock solution with Milli-Q water. This solution was then subjected to overnight stirring. The majority of emulsions were prepared by means of homogenizing 0.75 g of dextran solution (containing either 2 or 8 wt% silica) with 0.75 g of PEO solution containing PDADMAC at varying concentrations. This was accomplished by employing vortex mixing at 2500 rpm for 60 s. For some emulsions, the initial media of the polyelectrolyte and silica particles were exchanged. In these cases, 0.75 g of dextran solution with varying PDADMAC concentrations was emulsified with 0.75 g of PEO solution (containing 2 wt% silica) using identical vortex mixing conditions. All samples were prepared and stored at ambient temperature (23 °C).

### 2.4. Fluorescence Imaging

Two types of investigations were carried out. In the initial case, a microscopic slide equipped with a cavity-well was utilized. A drop of emulsion was placed into the well, which was then immediately closed with a cover slip without making contact with the emulsion sample. In the second method, a small emulsion drop was placed on a standard microscopic slide. Then, a cover slip was placed directly on top of the emulsion drop. This resulted in the rapid spreading of the emulsion sample between the cover slip and the glass slide. In each instance, the emulsions were examined following a 24 h period of synthesis. The imaging of each sample was conducted using a fluorescent microscope (Nikon Eclipse E600 equipped with a mercury lamp; Nikon, Tokyo, Japan) and a monochrome camera (Andor Zyla 5.5, Oxford Instrument Technology, Abingdon, UK). The images were captured using a 20× fluorescent objective and the NIS Elements (Nikon) software. The optical system comprised a mercury lamp white light source and a Nikon FITC filter set, which included an excitation filter of 480/30 nm, a dichroic mirror of 505 nm, and a barrier filter of 535/40 nm. All image exposures were optimized for optimal visibility using a live intensity spectrum.

### 2.5. Rheological Measurements

The shear-rate dependence of the viscosity of the separated top and bottom equilibrium aqueous phases at a total composition of 5.4 wt% PEO and 11.1 wt% dextran for each of the three investigated ATPSs was measured using a Malvern m-VROCi Microfluidic Rheometer. For the purpose of comparison, the shear-rate-dependent viscosities of pure 10.8 wt% PEO and 22.2 wt% dextran solutions were also determined. The ATPSs were prepared by homogenizing 0.75 g of 22.2 wt% dextran solution with 0.75 g of 10.8 wt% PEO solution using vortex mixing at 2500 rpm for 60 s, followed by centrifugation at 20,000 rpm for 15 min using Hettich Mikro 200R microcentrifuge. The collection of the separated phases was executed through the utilization of a 1 mL Hamilton syringe. Viscosity measurements were performed with the Malvern m-VROCi rheometer, which was equipped with a 13RA05100430-type sensor (flow channel gap: 20 μm, maximum pressure: 10,000 μPa) and a ThermoCube high-precision thermostat. All measurements were conducted within a temperature range of 25.0 ± 0.1 °C. The applied flow rates were adjusted to encompass the full available pressure range of the sensor. Specifically, flow rates of 1.0, 1.5, 3.0, and 5.0 μLmin^−1^ were utilized for the separated top phases and for the pure 10.8 wt% PEO solutions containing either the PEO 20 kDa or the PEO 100 kDa samples, as well as for the bottom phases and for the pure 22.2 wt% dextran solution. In the case of PEO 4 kDa, flow rates of 10.0, 15.0, 25.0, and 30.0 μLmin^−1^ were applied upon measuring the viscosity of the top phase or the 10.8 wt% PEO solution. The measurement times were set to 300 s (s). For each sample, at least three parallel measurements were performed, and all samples were prepared in triplicate. Between each sample, the sensor was meticulously rinsed with Milli-Q water until the measured viscosity of water was attained.

### 2.6. Electrophoretic Mobility Measurements

The mean electrophoretic mobility of silica particles and their aggregates with PDADMAC was measured using a Zetasizer Nano ZSP (Malvern Panalytical, Worcestershire, UK) instrument. A total of 4 mL of silica/PDADMAC dispersion was prepared according to the following procedure: First, 2 mL of silica dispersion with the desired concentration (2 wt%) was prepared and stirred at 600 rpm with a magnetic stirrer. Subsequently, 2 mL of PDADMAC solution with a defined concentration (0.00–0.18 wt%) was added to the silica dispersion using an automatic pipette under continuous stirring. Then, the samples were further stirred for a 15 s mixing period and subsequently transferred into a DTS1070 folded capillary cell utilizing a syringe. Electrophoretic mobility measurements were conducted at 25 °C, with a minimum of three parallel measurements performed for each concentration. The mean particle velocity (*v*_E_) at a given electric field strength (E) was determined based on the M3-PALS technique from the frequency shift of the scattered light caused by particle motion. The mean electrophoretic mobility (*u*_ζ_) was calculated using the relationship *u*_ζ_ = *v*_E_/E.

### 2.7. Interfacial Tension Measurements

A spinning drop tensiometer (KRÜSS) was utilized to measure the interfacial tension between the PEO-rich and dextran-rich aqueous phases that were formed after the centrifugation of the PEO/dextran aqueous mixtures. A drop of the PEO-rich phase (approximately 0.6–0.8 µL) was injected into a cup that was positioned in proximity to a cuvette containing the dextran-rich phase (approximately 0.4 mL). During the measurement process, the cuvette undergoes a rotational movement. The rate of rotation can be modified to control the rate at which a drop detaches from the cup and enters the cuvette. The movement and shape of the drops are monitored by a camera, and the interfacial tension is determined from the contour of the drops using the Vonnegut equation and the ADVANCE software belonging to the instrument. The measurements were conducted within a temperature range of 25.0 ± 0.1 °C, employing a Thermo Scientific thermostat. The operational rotation speed was configured to 4000 rpm. The density values of the polymer solutions, which are necessary for the calculation of the interfacial tension data, were determined at 25.00 ± 0.01 °C using an Anton Paar DMA 60 instrument. A more thorough exposition on the methodology employed in the density measurements can be found in the Supplementary Materials.

## 3. Results and Discussion

### 3.1. Characterization of PEO/Dextran ATPS Systems

**Figure 1:**
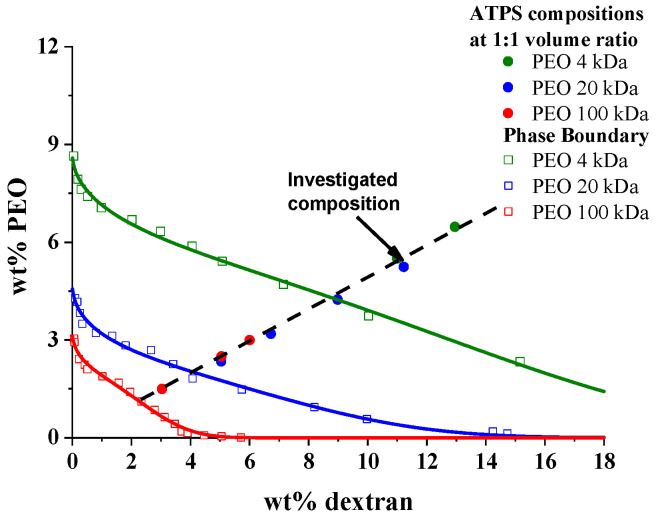
The phase diagrams for aqueous mixtures of dextran and PEO with average molecular weights of 4 kDa, 20 kDa, and 100 kDa, respectively. The dashed line connects those compositions within the two-phase regions where the volumes of the top (PEO-rich) and bottom (dextran-rich) phases are equal within experimental errors. To connect the experimental points of the binodals, they were fitted with an empirical equation [33,36] of c_PEO_ = c_1_ × exp(−c_2_ × [c_DEX_]^1/2^ × c_3_ × [c_DEX_]^3^), where c_PEO_ and c_DEX_ are the concentrations of PEO and dextran in wt%, respectively, and c_1_, c_2_, and c_3_ are constants.

To study the impact of PEO molecular weight on the emulsification of PEO/dextran ATPSs, their phase properties must be examined. Figure 1 shows the experimental binodal curves of the PEO 4 kDa/dextran, PEO 20 kDa/dextran, and PEO 100 kDa/dextran mixtures. Consistent with previous findings, the phase boundaries are shifted to lower polymer concentrations, and the phase diagram becomes more symmetrical as the molecular weight of the PEO samples increases [33,34]. Because bijels are expected to form at equal volumes of immiscible phases [10,35], a few compositions with a 1:1 volume ratio within the two-phase regime were determined separately for the investigated PEO/dextran mixtures prepared with PEO samples of different molecular weights. As Figure 1 shows, these compositions have a unique characteristic: they fall on the same linear curve for all three ATPSs.

To systematically investigate the effect of polyethylene oxide molecular weight on emulsion structure and stability, a composition of 5.4 wt% PEO and 11.1 wt% dextran was chosen. A 10.8 wt% PEO solution was layered on top of an equal-mass 22.2 wt% dextran solution, and the mixture was homogenized for 60 s, followed by centrifugation for 15 min. At these polymer concentrations, the interfacial tension values (γ) were found to be 0.050 ± 0.005 mN/m for the PEO 4 kDa/dextran mixture, as well as 0.160 ± 0.005 and 0.140 ± 0.005 mN/m for the PEO 20 kDa/dextran and PEO 100 kDa/dextran mixtures, respectively (the density data of the separated PEO and dextran-rich phases are shown in Table S1 in the Supplementary Materials). These interfacial tension values agree with earlier measurements on ATPSs with similar compositions of PEO and dextran samples of nearly the same molecular weights as those used in this study [37].

**Figure 2:**
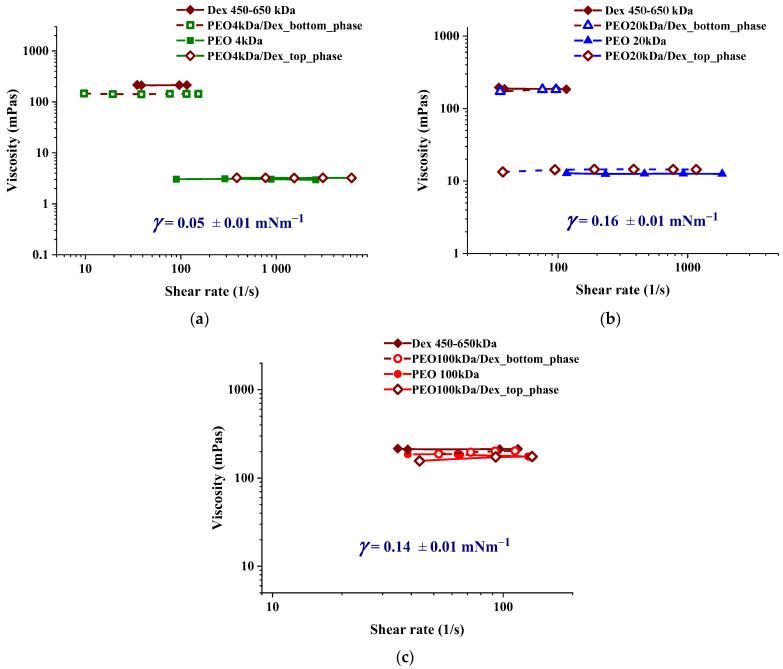
The shear-ratedependence of the viscosity of the separated bottom and top equilibrium aqueous phases at the total ATPS composition of 5.4 wt% PEO and 11.1 wt% dextran (open symbols). For comparison, the shear rate dependence of the viscosity values of pure 10.8 wt% PEO and 22.2 wt% dextran solutions are also shown (closed symbols): (**a**) PEO 4 kDa/dextran; (**b**) PEO 20 kDa/dextran; (**c**) PEO 100 kDa/dextran ATPSs. Additionally, the graphs show the interfacial tension values of the corresponding ATPSs, as measured by the spinning drop method.

Regarding emulsion stability, another important characteristic of the adjacent aqueous phases is their rheological behavior. Figure 2 shows the shear-rate-dependent viscosities of the separated top and bottom phases of the three investigated PEO/dextran ATPSs. As the graphs indicate, the viscosities of the top PEO-rich phases are two and one orders of magnitude lower than those of the dextran-rich bottom phases for the PEO 4 kDa and 20 kDa samples, respectively. For PEO 100 kDa/dextran mixtures, the separated aqueous phases have similarly high viscosities. For comparison, Figure 2 also plots the viscosities of the pure 10.8 wt% PEO and 22.2 wt% dextran solutions. All these polymer solutions exhibited Newtonian behavior, with viscosity values consistent with previous results on polyethylene oxide (PEO) and dextran solutions with comparable polymer concentrations and molecular weights [38,39,40]. As can be seen, the viscosities of the equilibrated top and bottom phases are similar to those of the initial pure polymer solutions, indicating that they are largely enriched in PEO and dextran, respectively.

### 3.2. Silica Particle/PDADMAC Association

**Figure 3:**
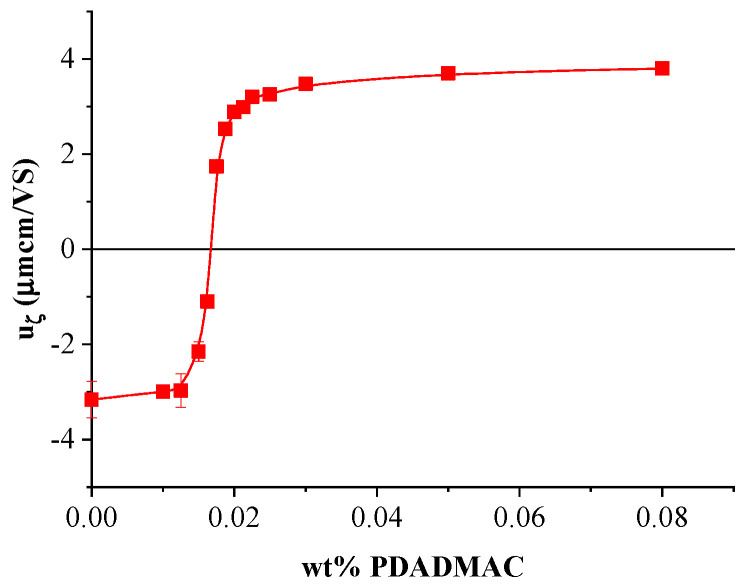
Mean electrophoretic mobility of PDADMAC-coated silica nanoparticle agglomerates (at 1 wt% silica) as a function of PDADMAC concentration.

The association of the cationic polyelectrolyte with silica particles was tested via electrophoretic mobility measurements at a constant silica concentration of 1 wt% and different polyelectrolyte concentrations, as shown in Figure 3. As expected, the polycations bound to the surface of the silica particles gradually compensate for and then neutralize their negative surface charge as the PDADMAC concentration increases [41,42]. At even higher polyelectrolyte concentrations, the increased adsorption of polyions leads to a charge inversion [41,42] of the particles. Figure S1 shows that at PDADMAC concentrations around charge neutralization (i.e., at c_PDADMAC_ = 0.015 wt%), adsorption of the cationic polyelectrolyte leads to fast coagulation. The formed agglomerates nearly completely sediment within an hour. Coagulation of moderately overcharged silica particles and subsequent sedimentation is slower in the presence of 0.030 wt% PDADMAC. At a significantly larger polyelectrolyte excess, i.e., at c_PDADMAC_ = 0.090 wt%, a turbid dispersion with only a small amount of sediment was visually observable even after one day, indicating the reduced aggregation and sedimentation rate of the polycation-coated silica particles due to excess charges of the adsorbed polyions.

### 3.3. Aging Processes in Emulsions with 1 and 4 wt% Silica Concentrations

**Figure 4:**
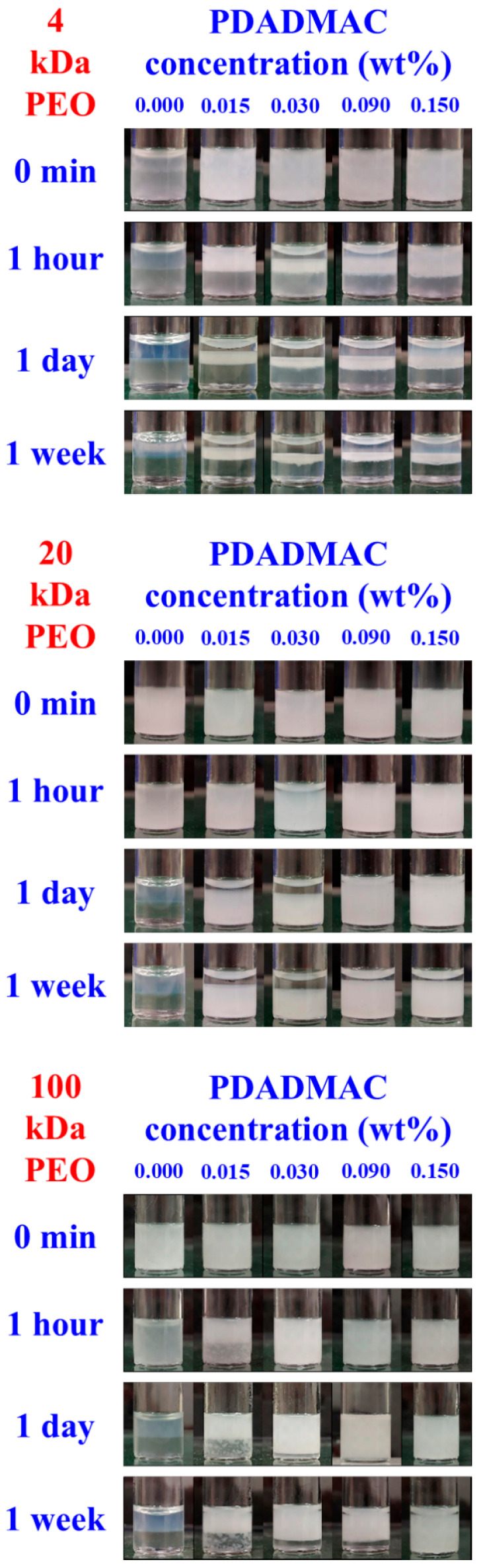
The visual appearance of emulsions over time in the presence of 1 wt% silica and different PDADMAC concentrations. **Top:** PEO 4 kDa/dextran; **middle:** PEO 20 kDa/dextran; **bottom:** PEO 100 kDa/dextran systems.

Emulsions were prepared using Vortex mixing over a wide PDADMAC-to-silica ratio range for the three types of ATPSs at a total composition of 5.4 wt% PEO and 11.1 wt% dextran. Specifically, a dispersion containing 2 wt% silica and 22.2 wt% dextran was homogenized with an equal mass of a 10.8 wt% PEO solution containing varying concentrations of PDADMAC. Figure 4 shows the evolution of the visual appearance of the emulsified systems over time for the three PEO/dextran systems, respectively, up to one week. Each emulsion system at a given PEO, dextran, PDADMAC, and silica concentration was prepared at least twice, and the observed trends in emulsion stability were consistent with those shown in Figure 4. Fluorescence microscopy images unambiguously revealed the formation of PEO-in-dextran emulsions for each investigated ATPS at all PDADMAC concentrations (see Figures S2 and S3 in the Supplementary Materials for a few examples).

The PEO 4 kDa/dextran system formed the least stable emulsions. In this case, rapid creaming was observable. Within a couple of hours at lower PDADMAC-to-silica ratios and within days at larger polyelectrolyte excesses, coalescence of the PEO-rich droplets led to nearly complete phase separation, leaving a thin emulsion layer concentrated in PDADMAC/silica precipitates in the middle phase. In the case of the PEO 20 kDa/dextran system, creaming was not observable, and separation occurred into a transparent top phase and an emulsion-like bottom phase. This process was fastest (within hours) at 0.030 wt% and slowest (within days) at 0.090 and 0.150 wt% PDADMAC concentrations.

For the PEO 100 kDa/dextran mixtures, the emulsion stability trends remarkably deviated from those observed with the lower molecular weight PEO samples. Fast creaming occurred within hours at 0.015 wt% PDADMAC concentration, and precipitates stuck to the glass wall in the bottom phase. In contrast, much slower creaming was detected at 0.030 and 0.090 wt% PDADMAC concentrations; no creaming occurred at the largest polyelectrolyte-to-silica ratio. Furthermore, except for the 0.015 wt% PDADMAC concentration, the emulsion systems were significantly more stable with respect to coalescence than those prepared from the PEO 4 kDa/dextran or PEO 20 kDa/dextran ATPSs.

**Figure 5:**
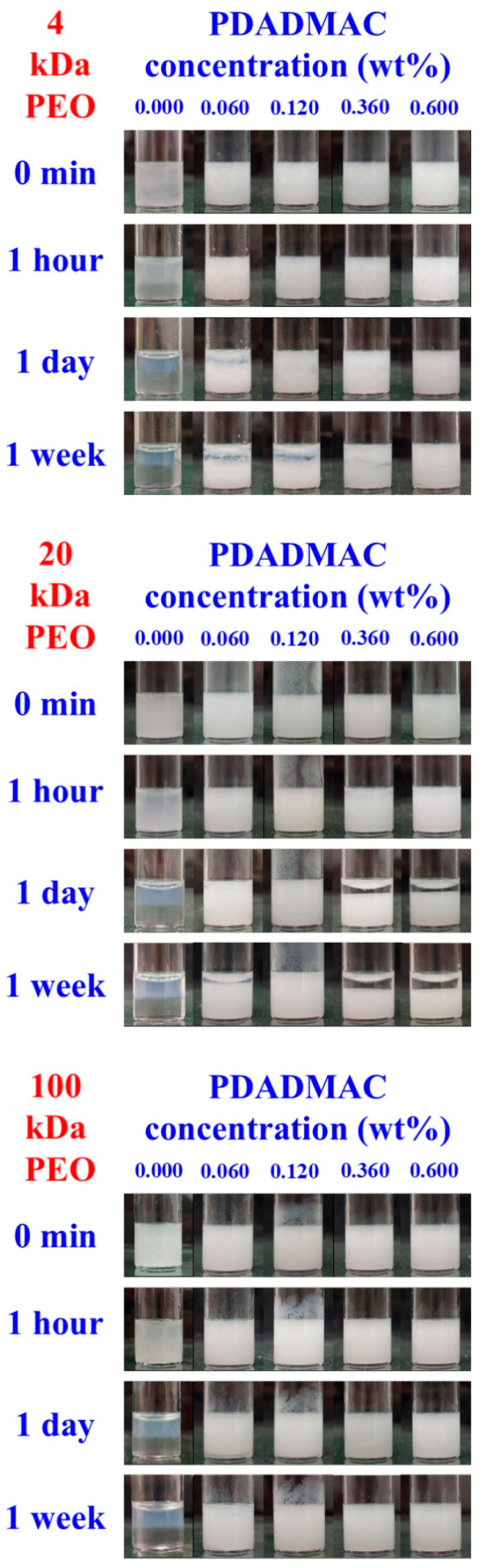
The visual appearance of emulsions over time in the presence of 4 wt% silica and different PDADMAC concentrations. **Top:** PEO 4 kDa/dextran; **middle:** PEO 20 kDa/dextran; **bottom:** PEO 100 kDa/dextran systems.

Figure 5 demonstrates the variation in the visual appearance of emulsions over time up to one week at concentrations of silica particles and PDADMAC four times larger than those in Figure 4. The emulsions were prepared by mixing equal masses of a silica dispersion in dextran solution (8 wt% silica and 22.2 wt% dextran) with a 10.8 wt% polyethylene oxide (PEO) solution containing varying concentrations of PDADMAC. As with the findings at a 1 wt% silica concentration, PEO-in-dextran emulsions formed at each composition, as demonstrated by fluorescence microscopy images taken at a 4 wt% silica concentration (see Figure S4 for a few examples).

At first glance, Figure 5’s results indicate that the PEO 4 kDa/dextran systems are much more stable, with reduced coalescence and creaming rates, compared to the observations at 1 wt% silica. However, closer inspection revealed heterogeneous emulsion systems with large amounts of precipitates and sediments visible to the naked eye at each investigated PDADMAC-to-silica ratio. The aging processes of the PEO 20 kDa/dextran emulsion systems also deviate from that observed at the lower silica concentration. At the lowest PDADMAC-to-silica ratio, slower coalescence was found than at the 1 wt% silica concentration, but no creaming was observed. At 0.12 wt% PDADMAC and 4 wt% silica, an emulsion highly stable against creaming and coalescence formed, unlike at 1 wt% silica, where fast coalescence but no creaming occurred. At the two largest PDADMAC-to-silica ratios, only coalescence was observable, similarly to the results for 1 wt% silica. The PEO 100 kDa/dextran system exhibited markedly different emulsion stability features compared to those observed at 1 wt% silica concentration. In sharp contrast to the emulsions prepared at the lower silica concentration, emulsions that were highly stable against both creaming and coalescence were formed at all investigated PDADMAC-to-silica ratios in the presence of 4 wt% silica. These emulsions remained stable for months.

### 3.4. Dependence of Emulsion Stability on PEO Molecular Weight

Pickering emulsions are stabilized by particle adsorption at the liquid–liquid interface, which depends on the adsorption driving force, the concentration of the particles, and the kinetics of adsorption [43]. However, the stabilization via interfacial membrane formation is a more complex phenomenon than the classical Pickering stabilization [17,22]. Hahn and colleagues revealed that the complexation of the oppositely charged silica particles and PDADMAC molecules, initially separated into adjacent aqueous phases, led to a (2–4 μm) thick, coherent solid layer at the water/water interface of a similar ATPS (with 10 wt% PEO 20 kDa and 15 wt% dextran 450–650 kDa) [32] to that investigated here. This interfacial film effectively stabilized all-aqueous double emulsion microcapsules in the given ATPS. Additionally, it facilitated the transportation of small molecules and PEO [32].

The aforementioned interfacial membrane forms through the adsorption of PDADMAC/silica aggregates. Since there are only moderate differences in the interfacial tension data of the three investigated PEO/dextran ATPSs, it can be hypothesized that the size of PEO molecules exerts a predominant influence on the stability of emulsions primarily through its effect on the mechanism of formation and adsorption of PDADMAC/silica agglomerates.

The formation of particle agglomerates is contingent upon several processes, including the transport of PDADMAC molecules and the migration of silica particles from the adjacent aqueous phases to the water/water (W/W) interface. The binding of PDADMAC to the oppositely charged nanoparticles subsequently leads to the coagulation of polycation-coated particles. It is likely that certain aggregates, which are formed during intermediate stages of local coagulation within the interfacial region, have the capacity to adsorb at the W/W interface. This underscores the critical importance of this coagulation process. Concurrent processes such as further aggregation and/or sedimentation of the formed agglomerates can markedly diminish the adsorbed amount of the polycation-coated particles, should the coagulation rate be excessively elevated. This, in turn, can impede the formation of a robust, stabilizing membrane. Conversely, if the local aggregation is too slow, then it results in the failure of the smaller particle aggregates to adsorb and form an interfacial film before the coalescence of the droplets. Furthermore, the ATPS-forming polymers may demonstrate a selective binding tendency towards the PDADMAC/silica aggregates, thereby modulating their capabilities in terms of interfacial film formation.

It is also important to note that interfacial membrane formation may be accompanied by concurrent rheological alterations within the PEO and dextran-rich phases. These include, for instance, bulk network formation in either of the liquid phases due to the non-adsorbed aggregates [43,44] or the various transport processes driven by the unequal chemical potentials of the different components in the dispersed and continuous phases of the emulsion [32]. In addition to the previously mentioned processes of interfacial film formation, these rheological changes also affect the creaming and coalescence of the PEO-rich droplets.

In the context of the PEO 4 kDa/dextran system, it is hypothesized that the aggregation rate of PDADMAC-coated silica particles in the proximity of the interfaces will be the most substantial among the investigated ATPS systems. This is attributed to the remarkably low viscosity of the PEO-rich phase, which concomitantly expedites the diffusion of PDADMAC molecules toward the interface and their subsequent adsorption onto silica. Consequently, rapid precipitation and sedimentation occur with minimal adsorption of PDADMAC/silica agglomerates of suitable size, resulting in rapid creaming and coalescence at 1 wt% silica concentration. As the particle concentration increases, the rates of adsorption and bulk coagulation concomitantly rise, thereby slightly enhancing the stability of the PEO 4 kDa/dextran emulsion systems, particularly at the largest PDADMAC-to-silica ratio, where the polycation-coated particles exhibit the most reduced aggregation rate.

In the case of PEO 20 kDa/dextran systems, the transport and adsorption of PDADMAC molecules, as well as the local coagulation of polycation-coated silica particles, are slower than in the PEO 4 kDa/dextran ATPS system due to the larger viscosity of the PEO-rich phase. While the adsorption of agglomerates with appropriate sizes might be higher than in the PEO 4 kDa/dextran emulsions, it is still not sufficient to prevent coalescence at a silica concentration of 1 wt%. In addition, the non-adsorbed and yet not sedimented agglomerates may induce network formation within the concentrated and highly viscous dextran-rich phase, which prevents creaming at the investigated PDADMAC-to-silica ratios, as demonstrated in Figure 4. The increase in particle concentration enhances both local coagulation and adsorption rates. The intricate interplay among these processes is pivotal in dictating the extent of particle agglomerate adsorption as well as the progression of non-adsorbed particle-induced network formation within the dextran-rich phase. The formed networks impede creaming and, under certain compositions, could even preclude the coalescence of droplets, as evidenced by observations at 0.12 wt% PDADMAC and 4 wt% silica concentrations.

In the case of the largest molecular weight PEO sample, both aqueous phases exhibit commensurably high viscosity. The association and subsequent aggregation of silica particles and PDADMAC molecules into larger agglomerates is the slowest among the three investigated ATPSs. Consequently, agglomerates within a suitable size range may have sufficient time to adsorb, thereby creating the thickest adsorbed layer in the PEO 100 kDa/dextran systems. Since most of the aggregates could adsorb at the W/W interface, the evolution of a network based on non-adsorbed particles is retarded in the dextran-rich phase. Consequently, contrary to the PEO 20 kDa/dextran system, the creaming of the PEO-rich droplets is not hindered at 1 wt% silica for the PEO 100 kDa/dextran system. The presence of a robust interfacial film surrounding the PEO-rich droplets results in the formation of emulsions with greater stability against coalescence in comparison to the other ATPSs. The sole exception was the PEO 100 kDa/dextran system at the lowest PDADMAC-to-silica ratio, where a rapid creaming process and the subsequent formation of bulk precipitates were observable within the dextran phase. This latter phenomenon can be attributed to the bridging flocculation of the neutral PDADMAC-coated silica particles through adsorbed PEO molecules on their surface, resulting in rapid aggregation and bulk precipitation. This observation aligns with the presence of sticky precipitates that can be observed on the glass wall of the vial at this composition in the presence of 1 wt% silica.

Finally, the formation of highly stable emulsions at 4 wt% silica, as evidenced by the absence of creaming or coalescence over an extended period of weeks, is primarily attributable to the presence of highly viscous dextran and PEO-rich phases. The enhanced viscosity of the aqueous phases attenuates both the coagulation and sedimentation of PDADMAC/silica agglomerates. Concurrently, the elevated particle concentration fosters enhanced adsorption, thicker interfacial film, and more pronounced network formation (due to the larger amount of non-adsorbed aggregates) in comparison to the PEO 100 kDa/dextran ATPSs at 1 wt% silica concentration.

### 3.5. The Effect of Starting Media

Since the PDAMAC and silica are soluble or dispersible in both the PEO and dextran-rich phases, it can be posited that their initial aqueous media, prior to emulsification, may also influence the aging of the emulsions. This hypothesis is supported by evidence from oil/water emulsions stabilized by particles of intermediate hydrophobicity [45,46]. Furthermore, the association of oppositely charged components has been demonstrated to manifest pronounced non-equilibrium characteristics and order of addition effects [47,48], particularly in the presence of non-ionic polymers [49].

**Figure 6:**
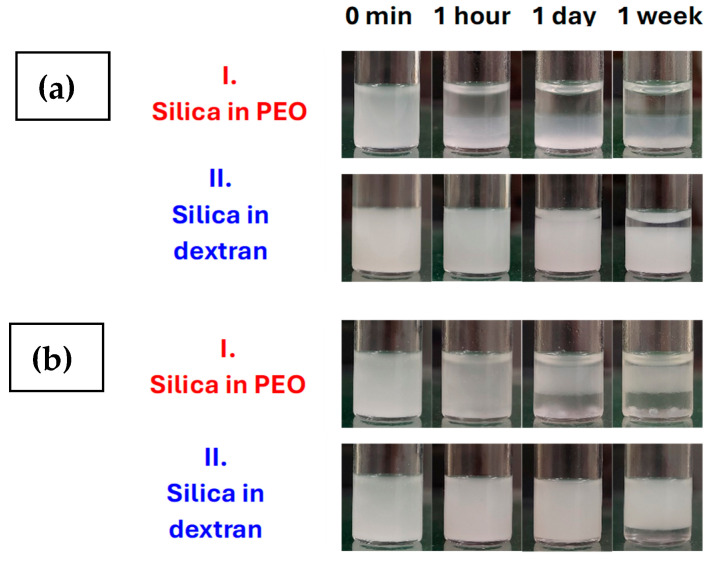
Variation in the visual appearances of the emulsions over time when prepared at the same total concentrations of components using two different protocols. In the first method, the silica was dispersed in the PEO solution, while PDADMAC was dissolved in the dextran solution. The two equal-mass systems were emulsified by vortex mixing. In the second method (II), the silica was dispersed in the dextran solution, and the PDADMAC was dissolved in the PEO solution. Then, these two aqueous systems of equal mass were mixed in the same way as in the first method. (**a**) PEO 20 kDa/dextran emulsions with 1 wt% silica and a 0.15 wt% PDADMAC concentration. (**b**) PEO 100 kDa/dextran emulsions with 1 wt% silica and a 0.09 wt% PDADMAC concentration.

Indeed, the impact of the initial polymer solution medium of PDADMAC and silica was found to be significant for some of the ATPSs with PEO 20 and 100 kDa samples, as illustrated in Figure 6. Specifically, when silica was placed in a PEO solution and PDADMAC in a dextran solution, a significant reduction in stability against both creaming and coalescence was observed compared to the results with the reverse scenarios at the same composition, i.e., with silica particles in the dextran solution and PDADMAC molecules in the PEO solution initially. The observed outcomes are ascribed to the binding of PEO molecules to the bare silica surface [50], a phenomenon that is significantly pronounced when silica particles contact the PEO molecules first. The preadsorbed PEO molecules could have a considerable effect on the binding of PDADMAC molecules to silica particles, which in turn affects their subsequent coagulation. In such instances, the initial interaction between silica particles and PEO molecules is likely to result in an increased propensity for bridging flocculation of the silica/PDADMAC complexes through adsorbed uncharged macromolecules. This phenomenon is particularly pronounced in the presence of larger PEO molecules. The largely accelerated bulk coagulation of silica/PDADMAC complexes impedes their adsorption process and the tendency of bulk network formation. This results in diminished stability when compared to analogous ATPSs with identical compositions, where the silica particles are located within the dextran solution prior to emulsification, as depicted in Figure 6.

### 3.6. Formation of Bicontinuous Structures Under Confinement

**Figure 7:**
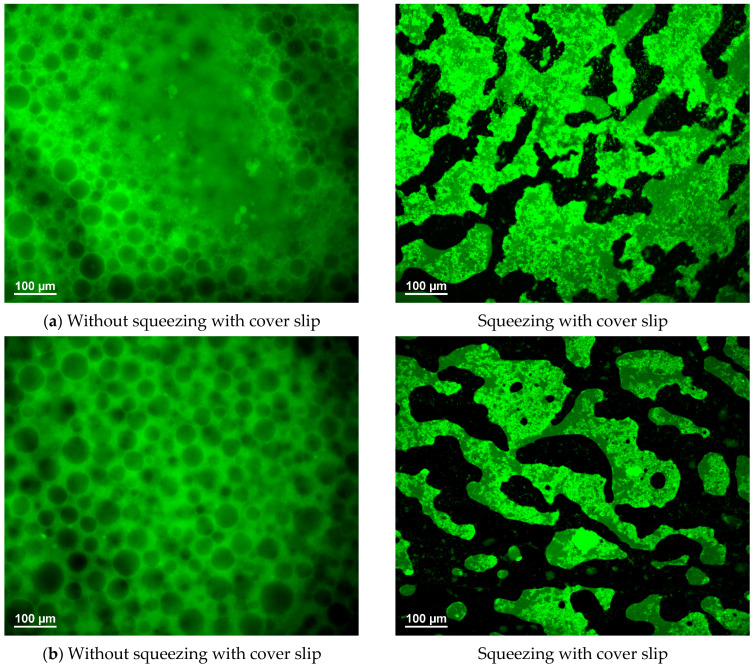
Fluorescence microscope images of the PEO 100 kDa/dextran emulsions taken one day after their formation in the presence of (**a**) 1 wt% silica and 0.030 wt% PDADMAC, (**b**) 1 wt% silica and 0.150 wt% PDADMAC. **Left:** without; **right:** with compressing by cover slip. The horizontal bar denotes 100 μm.

Another noteworthy finding emerged from microscopic investigations of the emulsions. Each emulsion sample was subjected to two distinct forms of analysis. In the initial case, an emulsion drop was placed into a designated cavity of a microscopic slide. Subsequently, a cover slip was placed onto the top of the well to prevent evaporation without contacting or deforming the drops. In the second case, following the injection of a minute emulsion drop onto a conventional microscopic slide, a cover slip was promptly positioned atop the drop. This resulted in the immediate spreading of the emulsion, which was subsequently confined to a thin layer between the slide and the cover slip. As demonstrated in Figure 7 and Figure S3 for the PEO 100 kDa/dextran ATPS, a PEO-in-dextran type of emulsion versus a bicontinuous emulsion structure was observed in the absence and presence of compression, respectively, for the same emulsions at 1 wt% silica concentration. Bicontinuous structures were not observed; instead, evidence of deformation and coalescence of the quasi-spherical PEO-rich droplets under compression was identified in emulsions comprising lower-molecular-weight PEO samples or silica concentrations of 4 wt%, as illustrated in Figures S2 and S4.

The formation of bijels is typically attributed to the arrest of spinodal decomposition in a homogeneous liquid mixture. This process occurs in the context of coarsening of liquid domains, which in turn leads to the jamming of adsorbed particles. In this manner, the emulsion system becomes entrapped within a bicontinuous structure, exhibiting a net zero average surface curvature of the liquid/liquid interface [9,10]. However, in certain systems, interpenetrating liquid domains have been observed to form subsequent to the straightforward mixing of immiscible phases [30,51,52]. In these instances, either the interfacial complexation of particles with polymers or the implementation of multistep preparation protocols involving highly viscous oil phases has been employed to trap a bicontinuous emulsion structure. The underlying mechanisms responsible for the development of bicontinuous domains in these systems remain to be fully elucidated. However, it is plausible to attribute this phenomenon to the phase inversion kinetics of the emulsions. During phase inversion, the net curvature of the liquid/liquid interface undergoes a transition from a convex to a concave configuration, thereby traversing the zero curvature point. In the event that the system composition is characterized by phase inversion and the experimental parameters are conducive to ensuring the appropriate timing for the adsorption of stabilizing particles or interfacial complexes, the jamming of these particles may arrest the bicontinuous domains that are transiently established during the phase inversion process.

In all the aforementioned reports, non-invasive microscopic sampling methods were employed without the application of a coverslip on the top of the emulsion sample. Conversely, the images on the right side of Figure 7 and Figure S3 illustrate the formation of a bicontinuous-like emulsion structure upon compressing the emulsion samples within a thin layer between the coverslip and the microscopic slide. The impact of capillary pressure on emulsion structure has scarcely been documented. In a study on W/O/W multiple emulsions [53], Jiao et al. revealed that this type of compression led to coalescence of the internal water droplets within the oil droplets, which was usually followed by their subsequent release into the external aqueous phase. At some specific concentrations of the stabilizers, however, the enlarged internal water droplets remained attached within the oil droplets through an elastic film formed between the internal and outer aqueous phases. However, the transformation of a system of quasi-spherical droplets into a bicontinuous structure upon compression, as illustrated in Figure 7 and Figure S3, remains unreported, irrespective of the emulsion system type.

Surface and wetting-induced bijel formations have been previously reported in systems where the spinodal phase separation occurred at different compositions of the initially homogeneous liquid mixtures under confinement as compared to the bulk case [54,55]. Our results demonstrate the combined effects of capillary pressure and confinement. Specifically, compressing the emulsion causes PEO-rich droplets to coalesce, which can lead to local phase inversion within the confined emulsion sample. The net zero curvature could be trapped through jamming of the adsorbed PDADMAC/silica particles utilizing the highly viscous nature of the PEO 100 kDa and dextran-rich phases. This phenomenon is absent in the ATPS systems comprising 4 kDa and 20 kDa PEO samples (see Figure 5) due to the significant reduction in viscosity exhibited by the PEO-rich phases in comparison to the PEO 100 kDa/dextran mixtures. Furthermore, the bijel-like structures were not observed even for the PEO 100 kDa/dextran emulsions in the presence of 4 wt% silica. This is likely attributable to the thick adsorbed layer and/or increased network formation, which hindered coalescence and resulted in elongated, anisometric emulsion droplets upon compression, as evidenced in Figure S4.

## 4. Conclusions

This study examined three ATPS systems that used the same high-molecular-weight dextran (450–650 kDa) and three poly(ethylene oxide) (PEO) samples with relative molecular weights of 4 kDa, 20 kDa, and 100 kDa. To facilitate comparison, we used a single composition of PEO/dextran mixtures with equal volumes of the aqueous phases to prepare emulsions stabilized by interfacial complexation of negatively charged silica particles with PDADMAC molecules. Our results show that the impact of PEO molecule size on emulsion aging is primarily manifested through its effect on the interfacial layer formation mechanism.

The specific interaction of PEO with the bare silica surface can cause bridging flocculation of polycation-coated silica particle aggregates, which significantly hinders their adsorption and reduces emulsion stability when the silica particles are initially located in a PEO solution. In the reverse scenario, the PDADMAC molecules and silica particles are separated in polyethylene oxide and dextran solutions, respectively. In this case, the impact of PEO molecular weight is primarily manifested through polymer solution viscosity. At the chosen ATPS composition, the viscosity of the PEO-rich phase is two orders of magnitude greater for the PEO 100 kDa/dextran mixture than for the PEO 4 kDa/dextran system. In the former case, both aqueous phases have similarly large viscosities, which markedly attenuate the formation and subsequent local coagulation of PDADMAC-coated silica particles. This enables adsorption and the formation of a stabilizing interfacial membrane. Thus, the most stable emulsions were observed for PEO 100 kDa/dextran mixtures, particularly at the highest silica concentration. At this concentration, the amount of adsorbed agglomerates and the network-forming capability due to non-adsorbed aggregates are both enhanced.

An interesting impact of the microscopic investigation method on emulsion structure was also found for PEO 100 kDa/dextran ATPSs at 1 wt% silica concentration and various PDADMAC-to-silica ratios. Compressing the emulsion sample through the capillary pressure between a coverslip and a microscope slide produced bicontinuous structures. In contrast, PEO-rich droplets in dextran were observable in the same emulsion sample when investigated using a noninvasive microscopic method without compression. We rationalized this finding by considering the high viscosities of the two aqueous phases, which could trap a bicontinuous structure formed by compression-induced phase inversion in PEO-in-dextran emulsions.

The results of our study could be used to optimize the molecular weight of ATPS-constituting polymers to synthesize all-aqueous emulsions with versatile properties. Further investigating the impact of the molecular architecture and/or hydrophilicity of these polymers on emulsion stability would also be desirable.

## Data Availability

The original contributions presented in this study are included in the article/Supplementary Materials. Further inquiries can be directed to the corresponding authors.

## Supplementary Materials

The following supporting information can be downloaded at https://www.mdpi.com/article/10.3390/polym17172305/s1. Details of density measurements. Table S1. Density data of the separated phases of PEO/dextran ATPSs. Figure S1. Sedimentation of the agglomerates of PDADMAC-coated silica particles at 1 wt% silica concentration. Figure S2. Fluorescence microscope images of the PEO 20 kDa/dextran emulsions in the presence of 1 wt% silica and different PDADMAC concentrations. Figure S3. Fluorescence microscope image of the PEO 100 kDa/dextran emulsion in the presence of 1 wt% silica and 0.090 wt% PDADMAC. Figure S4. Fluorescence microscope images of the PEO 100 kDa/dextran emulsions in the presence of 4 wt% silica and 0.36 wt% PDADMAC.

## References

[1] Pereira T.F., Borchardt H., Wanderley W.F., Vasconcelos U., Itamara F., Leite I.L. (2025). Pequi Pulp (*Caryocar brasiliense*) Oil-Loaded Emulsions as Cosmetic Products for Topical Use. Polymers.

[2] Wang L., Lin L., Li J., Liao J., Li B., Jiao W., Guo S. (2024). In Vitro Gastrointestinal Digestion of Corn-Oil-in-Water Pickering Emulsions: Influence of Lignin-Containing Cellulose Nanofibrils Loading. Polymers.

[3] Wei D., Yin N., Xu D., Ge L., Guo R. (2024). Dynamically Reconfigurable Complex Emulsion Droplets as Intelligent Microreactors. ACS Sustain. Chem. Eng..

[4] Gao X., Hou L., Yang W., Dong L., Ge X. (2025). Lignin-Based Nanoparticles Stabilized Pickering Emulsion for Enhanced Catalytic Hydrogenation. Langmuir.

[5] Johnson E., Koh A. (2024). Recent Advances in Smart Emulsion Materials: From Synthesis to Applications. Adv. Eng. Mater..

[6] Cheng Y., Xie J., Lu Y., Tian W., Wu T., Chen F., Gao W., Jin Y., Yuan L., Wang B. (2024). Interfacial Pickering Emulsion Polycondensation for Degradable Nanocomposites. ACS Macro. Lett..

[7] Zhao C., Wang X., Zhang J., Liu Y., Liu C., Huang B., Yang Y. (2024). Development and Application of High-Internal-Phase Water-in-Oil Emulsions Using Amphiphilic Nanoparticle- Based Emulsifiers. Polymers.

[8] Liang C., Qi N., Zhao L., Li X., Li Z. (2025). Development of a Water-Sensitive Self-Thickening Emulsion Temporary Plugging Diverting Agent for High-Temperature and High-Salinity Reservoirs. Polymers.

[9] Di Vitantonio G., Wang T., Stebe K.J., Lee D. (2021). Fabrication and Application of Bicontinuous Interfacially Jammed Emulsions Gels. Appl. Phys. Rev..

[10] Ghaffarkhah A., Hashemi S.A., Isari A.A., Panahi-Sarmad M., Jiang F., Russell T.P., Rojas O.J., Arjmand M. (2024). Chemistry, Applications, and Future Prospects of Structured Liquids. Chem. Soc. Rev..

[11] Thorson T.J., Gurlin R.E., Botvinick E.L., Mohraz A. (2019). Bijel-Templated Implantable Biomaterials for Enhancing Tissue Integration and Vascularization. Acta Biomater..

[12] Kharal S.P., Haase M.F. (2022). Centrifugal Assembly of Helical Bijel Fibers for PH Responsive Composite Hydrogels. Small.

[13] Cai D., Richter F.H., Thijssen J.H.J., Bruce P.G., Clegg P.S. (2018). Direct Transformation of Bijels into Bicontinuous Composite Electrolytes Using a Pre-Mix Containing Lithium Salt. Mater. Horiz..

[14] Ching H., Thorson T.J., Paul B., Mohraz A. (2021). Rapid Production of Bicontinuous Macroporous Materials Using Intrinsically Polymerizable Bijels. Mater. Adv..

[15] Neves B.S., Gonçalves R.C., Mano J.F., Oliveira M.B. (2024). Controlling the Diffusion of Small Molecules from Matrices Processed by All-Aqueous Methodologies: Towards the Development of Green Pharmaceutical Products. Green Chem..

[16] Gonçalves R.C., Oliveira M.B., Mano J.F. (2024). Exploring the Potential of All-Aqueous Immiscible Systems for Preparing Complex Biomaterials and Cellular Constructs. Mater. Horiz..

[17] Esquena J. (2023). Recent Advances on Water-in-Water Emulsions in Segregative Systems of Two Water-Soluble Polymers. Curr. Opin. Food Sci..

[18] Singla M., Sit N. (2023). Theoretical Aspects and Applications of Aqueous Two-Phase Systems. ChemBioEng Rev..

[19] Zhang J., Xie Y., Liu C., Cao H., Li Y., Li B., Zhang Y., Liu S. (2024). Water-in-Water Pickering Emulsion: A Fascinating Microculture Apparatus for Embedding and Cultivation of Lactobacillus Helveticus. Food Hydrocoll..

[20] Wei Y., Cai Z., Liu Z., Liu C., Kong T., Li Z., Song Y. (2024). All-Aqueous Synthesis of Alginate Complexed with Fibrillated Protein Microcapsules for Membrane-Bounded Culture of Tumor Spheroids. Carbohydr. Polym..

[21] Wang Q., Karadas Ö., Rosenholm J.M., Xu C., Näreoja T., Wang X. (2024). Bioprinting Macroporous Hydrogel with Aqueous Two-Phase Emulsion-Based Bioink: In Vitro Mineralization and Differentiation Empowered by Phosphorylated Cellulose Nanofibrils. Adv. Funct. Mater..

[22] Fick C., Khan Z., Srivastava S. (2023). Interfacial Stabilization of Aqueous Two-Phase Systems: A Review. Mater. Adv..

[23] Waldmann L., Nguyen D.-N.-T., Arbault S., Nicolai T., Benyahia L., Ravaine V. (2024). Tuning the Bis-Hydrophilic Balance of Microgels: A Tool to Control the Stability of Water-in-Water Emulsions. J. Colloid Interface Sci..

[24] Zhou C., Xie Y., Li Y., Li B., Zhang Y., Liu S. (2023). Water-in-Water Emulsion Stabilized by Cellulose Nanocrystals and Their High Enrichment Effect on Probiotic Bacteria. J. Colloid Interface Sci..

[25] Martin D., Buzza A., Fletcher P.D.I., Georgiou T.K., Ghasdian N. (2013). Water-in-Water Emulsions Based on Incompatible Polymers and Stabilized by Triblock Copolymers-Templated Polymersomes. Langmuir.

[26] Cui W., Xia C., Xu S., Ye X., Wu Y., Cheng S., Zhang R., Zhang C., Miao Z. (2023). Water-in-Water Emulsions Stabilized by Self-Assembled Chitosan Colloidal Particles. Carbohydr. Polym..

[27] Xie Y., Liu C., Zhang J., Li Y., Li B., Liu S. (2024). Crosslinking Alginate at Water-in-Water Pickering Emulsions Interface to Control the Interface Structure and Enhance the Stress Resistance of the Encapsulated Probiotics. J. Colloid Interface Sci..

[28] Murray B.S., Phisarnchananan N. (2014). The Effect of Nanoparticles on the Phase Separation of Waxy Corn Starch+locust Bean Gum or Guar Gum. Food Hydrocoll..

[29] Ma Q., Song Y., Kim J.W., Choi H.S., Shum H.C. (2016). Affinity Partitioning-Induced Self-Assembly in Aqueous Two-Phase Systems: Templating for Polyelectrolyte Microcapsules. ACS Macro Lett..

[30] Shekhar C., Kiran A., Mehandia V., Dugyala V.R., Sabapathy M. (2021). Droplet–Bijel–Droplet Transition in Aqueous Two-Phase Systems Stabilized by Oppositely Charged Nanoparticles: A Simple Pathway to Fabricate Bijels. Langmuir.

[31] Zhu Y., Beaumont M., Solin K., Spiliopoulos P., Zhao B., Tao H., Kontturi E., Bai L., Rojas O.J. (2024). Interfacial Membranization of Regenerated Cellulose Nanoparticles and a Protein Renders Stable Water-in-Water Emulsion. Small.

[32] Hann S.D., Stebe K.J., Lee D. (2017). AWE-Somes: All Water Emulsion Bodies with Permeable Shells and Selective Compartments. ACS Appl. Mater. Interfaces.

[33] Mistry S.L., Kaul A., Merchuk J.C., Asenjo J.A. (1996). Mathematical Modelling and Computer Simulation of Aqueous Two-Phase Continuous Protein Extraction. J. Chromatogr. A.

[34] Atefi E., Fyffe D., Kaylan K.B., Tavana H. (2016). Characterization of Aqueous Two-Phase Systems from Volume and Density Measurements. J. Chem. Eng. Data.

[35] Stratford K., Adhikari R., Pagonabarraga I., Desplat J.-C., Cates M.E. (2005). Colloidal Jamming at Interfaces: A Route to Fluid-Bicontinuous Gels. Science.

[36] Merchuk J.C., Andrews B.A., Asenjo J.A. (1998). Aqueous Two-Phase Systems for Protein Separation: Studies on Phase Inversion. J. Chromatogr. B.

[37] Ryden J., Albertsson P.-A. (1971). Interfacial Tension of Dextran-Polyethylene Glycol-Water Two-Phase Systems. J. Colloid Interface Sci..

[38] Tirtaatmadja V., Dunstan D.E., Boger D.V. (2001). Rheology of dextran solutions. J. Non-Newton. Fluid Mech..

[39] Ebagninin K.W., Benchabane A., Bekkour K. (2009). Rheological characterization of poly(ethylene oxide) solutions of different molecular weights. J. Colloid Interface Sci..

[40] Gonzalez-Tello P., Camacho F., Blazquez G. (1994). Density and Viscosity of Concentrated Aqueous Solutions of Polyethylene Glycol. J. Chem. Eng. Data.

[41] Hierrezuelo J., Sadeghpour A., Szilágyi I., Vaccaro A., Borkovec M. (2010). Electrostatic Stabilization of Charged Colloidal Particles with Adsorbed Polyelectrolytes of Opposite Charge. Langmuir.

[42] Voigt U., Jaeger W., Findenegg G.H., von Klitzing R. (2003). Charge Effects on the Formation of Multilayers Containing Strong Polyelectrolytes. J. Phys. Chem. B.

[43] Binks B.P. (2017). Colloidal Particles at a Range of Fluid–Fluid Interfaces. Langmuir.

[44] Ben Ayed E., Cochereau R., Dechancé C., Capron I., Nicolai T., Benyahia L. (2018). Water-In-Water Emulsion Gels Stabilized by Cellulose Nanocrystals. Langmuir.

[45] Ramos D.M., Sadtler V., Marchal P., Lemaitre C., Niepceron F., Benyahia L., Roques-Carmes T. (2023). Particles’ Organization in Direct Oil-in-Water and Reverse Water-in-Oil Pickering Emulsions. Nanomaterials.

[46] Binks B.P., Lumsdon S.O. (2000). Effects of Oil Type and Aqueous Phase Composition on Oil–Water Mixtures Containing Particles of Intermediate Hydrophobicity. Phys. Chem. Chem. Phys..

[47] Guzmán E., Maestro A., Ortega F., Rubio R.G. (2023). Association of Oppositely Charged Polyelectrolyte And Surfactant In Solution: Equilibrium and Nonequilibrium Features. J. Phys. Condens. Matter.

[48] Bali K., Varga Z., Kardos A., Mészáros R. (2019). Impact of Local Inhomogeneities on the Complexation between Poly(Diallyldimethylammoniumchloride) and Sodium Dodecyl Sulfate. Colloids Surf. A.

[49] Pojják K., Fegyver E., Mészáros R. (2013). Effect of Linear Nonionic Polymer Additives on the Kinetic Stability of Dispersions of Poly(Diallyldimethylammonium Chloride)/Sodium Dodecylsulfate Nanoparticles. Langmuir.

[50] Mathur S., Moudgil B.M. (1997). Adsorption Mechanism(s) of Poly (Ethylene Oxide) on Oxide Surfaces. J. Colloid Interface Sci..

[51] Zhang L., Tian Y., Song A., Hao J. (2023). Particle–Polymer Union with Changeable Wettability for Constructing Bijels Using a Simple Mixing Method. Langmuir.

[52] Macmillan K.A., Royer J.R., Morozov A., Joshi Y.M., Cloitre M., Clegg P.S. (2019). Rheological Behavior and in Situ Confocal Imaging of Bijels Made by Mixing. Langmuir.

[53] Jiao J., Rhodes D.G., Burgess D.J. (2002). Multiple Emulsion Stability: Pressure Balance and Interfacial Film Strength. J. Colloid Interface Sci..

[54] Clegg P.S., Herzig E.M., Schofield A.B., Egelhaaf S.U., Horozov T.S., Binks B.P., Cates M.E., Poon W.C.K. (2007). Emulsification of Partially Miscible Liquids Using Colloidal Particles: Nonspherical and Extended Domain Structures. Langmuir.

[55] Gam S., Corlu A., Chung H.-J., Ohno K., Hore M.J.A., Composto R.J. (2011). A Jamming Morphology Map of Polymer Blend Nanocomposite Films. Soft Matter.

