# Supplementary material for: Impact of Polymer Molecular Weight on Aging of Poly(Ethyleneoxide)/Dextran All-Aqueous Emulsions Stabilized by Oppositely Charged Nanoparticle/Polyelectrolyte Assemblies
Source: Polymers (Basel). 2025 Aug 26;17(17):2305. doi: 10.3390/polym17172305 (PMC12431505; doi:10.3390/polym17172305)
Supplement: Supplementary file 1 [file polymers-17-02305-s001.zip › polymers-3821303-supplementary.pdf]

Impact of polymer molecular weight on aging of poly(ethyleneoxide)/dextran all-aqueous emulsions stabilized by oppositely charged nanoparticle/polyelectrolyte assemblies

Attila Kardos[1,2], Mónika Bak[1], Emese Kovács[2], György Juhász[2], Mihály Cserepes[3], József Tóvári[3], Róbert Mészáros[1,2]

[1] Eötvös Loránd University, Institute of Chemistry, H-1117 Budapest, Pázmány Péter sétány 1/A, Hungary

[2] Selye J. University, Department of Chemistry, 945 01 Komarno, Slovakia

[3] National Institute of Oncology, Department of Experimental Pharmacology and the National Tumor Biology Laboratory, Ráth György u. 7, H-1122 Budapest, Hungary

Presenting author e-mail: rmeszaros@caesar.elte.hu

Results of the density measurements of separated phases of PEO/dextran ATPSs prepared with different molecular weight PEOs

Density measurements were performed using an Anton Paar DMA system, consisting of a DMA 602 density measuring cell and a DMA 60 density meter. All measurements were conducted at a temperature of 25.00 ± 0.01°C. Thermal equilibrium was maintained using two high-precision thermostats connected in series, and the ambient temperature was consistently controlled at 25°C. To ensure reliable and repeatable results, the measuring cell was filled using a New Era NE-4000 dual syringe pump. The stability of the instruments was confirmed by measuring the density of water in the samples on multiple occasions. Three sets of ATPS were prepared by mixing 4 grams of a 22.2 wt% dextran solution with 4 grams of a 10.8 wt% PEO solution (molecular weights of PEO: 4 kDa, 20 kDa, and 100 kDa) using vortex mixer at 2500 rpm for 60 seconds. The resulting emulsions were then separated by ultracentrifugation using a Beckman Coulter Optima XPN-100 at 25°C. Centrifugation was carried out at 50,000 rpm for a duration of one hour. To circumvent the concentration fluctuation that is introduced during the process of centrifugation, the separated phases were vortexed again prior to measurement and were equilibrated for 24 hours at 25°C.

Table S1. Density data of the separated top (PEO rich) and bottom (dextran rich) phases

	**4 kDa PEO/Dex - Top Phase**	**4 kDa PEO/Dex - Bottom Phase**
**density (ρ) with 5k period time**	**1.015016**	**1.078360**
propagation of error (Δρ)	1.821316E-06	9.238571E-07
**density (ρ) with 20k period time**	**1.015013**	**1.078364**
propagation of error (Δρ)	7.806790E-07	7.189503E-07
	**20 kDa PEO/Dex - Top Phase**	**20 kDa PEO/Dex - Bottom Phase**
**density (ρ) with 5k period time**	**1.015701**	**1.086742**
propagation of error (Δρ)	1.310088E-06	1.342408E-06
**density (ρ) with 20k period time**	**1.015701**	**1.086740**
propagation of error (Δρ)	5.281125E-07	1.185214E-06
	**100 kDa PEO/Dex - Top Phase**	**100 kDa PEO/Dex - Bottom Phase**
**density (ρ) with 5k period time**	**1.014235**	**1.074821**
propagation of error (Δρ)	1.404359E-06	9.230731E-07
**density (ρ) with 20k period time**	**1.014236**	**1.074823**
propagation of error (Δρ)	5.607258E-07	9.921112E-07

Visual monitoring of the sedimentation of particle aggregates in water at different PDADMAC-to-silica ratios

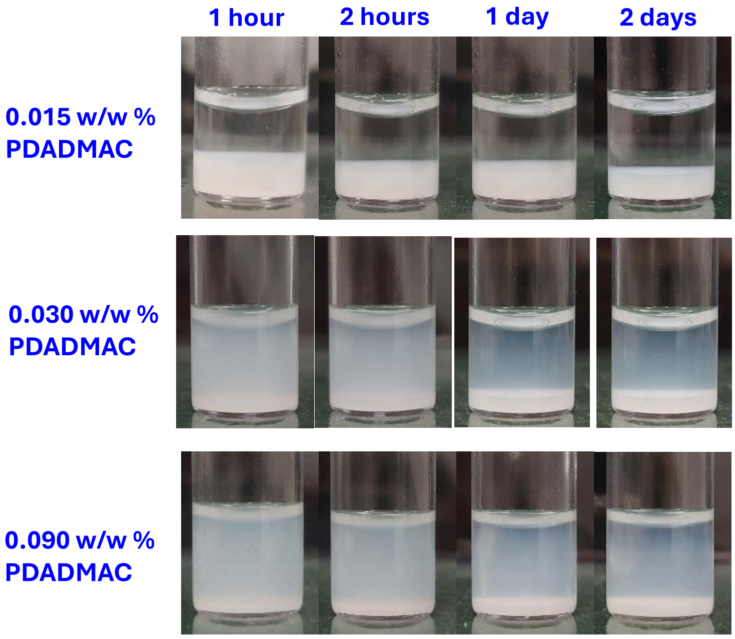

Figure S1. Sedimentation of the agglomerates of PDADMAC coated silica particles in the presence of 1 wt% silica concentration and different polyelectrolyte concentrations.

Fluorescence microscope images of PEO 20 kDa/dextran emulsion systems at 1 wt % silica

Two distinct protocols were implemented for microscopic investigations. In the initial method **(without squeezing with a cover slip)**, a drop of emulsion was deposited into the well of a specific microscopic slide that contained a cavity. This well was immediately sealed with a cover slip to prevent evaporation without coming into contact with the emulsion sample. In the second protocol (**with squeezing with cover slip**), following the deposition of a small emulsion sample onto a standard microscopic slide (without cavity well), a cover slip was positioned over the sample. This resulted in the rapid spreading of the emulsion into a thin layer, which spread across the cover slip and the glass slide. In each instance, the emulsions were examined following a 24-hour period of synthesis.

a.) Without squeezing with cover slip **Squeezing with coverslip**

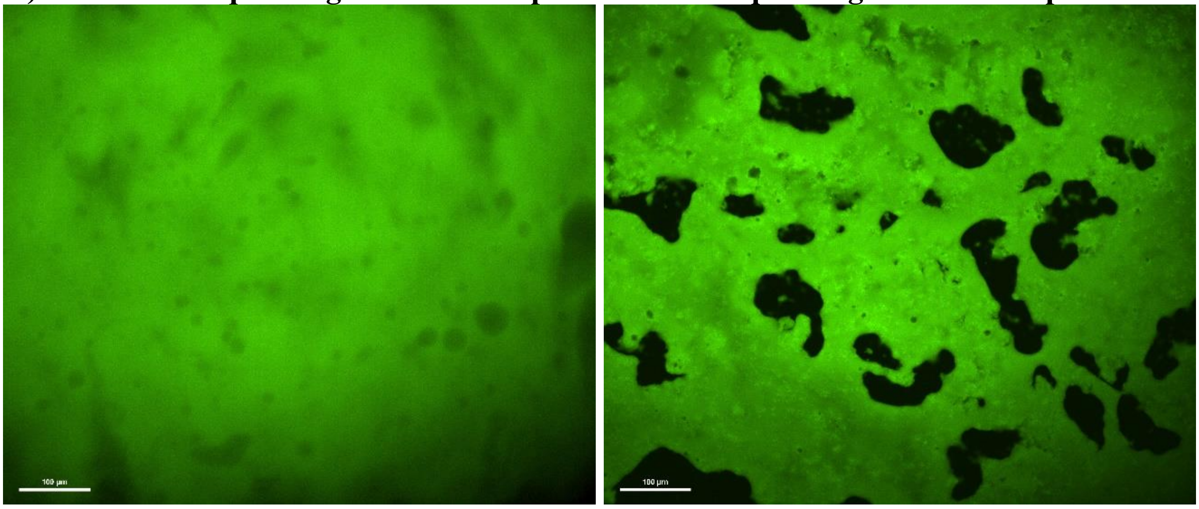

b.) Without squeezing with cover slip **Squeezing with coverslip**

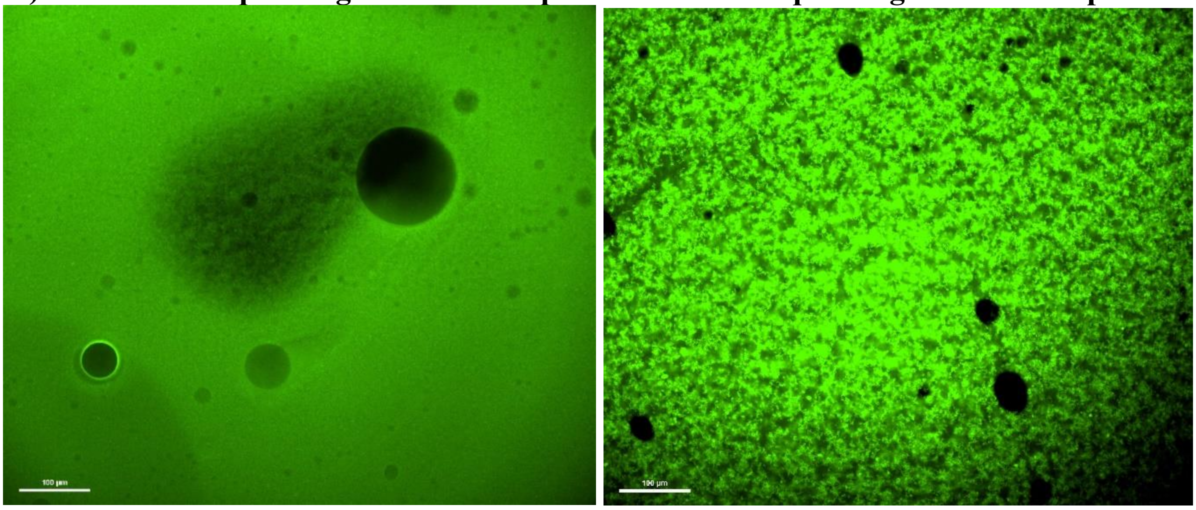

Figure S2. Fluorescence microscope images of the PEO 20 kDa/dextran emulsions (top phase) after one day after their preparation in the presence **a.)** 1 wt% silica and 0.030 wt% PDADMAC. and **b.)** of 1 wt% silica and 0.090 wt% PDADMAC. ***Left:*** without; ***right:*** with pressing by cover slip.

Fluorescence microscope images of PEO 100kDa/dextran emulsion systems at 1 wt % silica

The same two types of protocols as described previously for Figure S2 were applied during microscopic investigations. In each case, the emulsions were studied after one day of their synthesis.

Without squeezing with cover slip **Squeezing with coverslip**

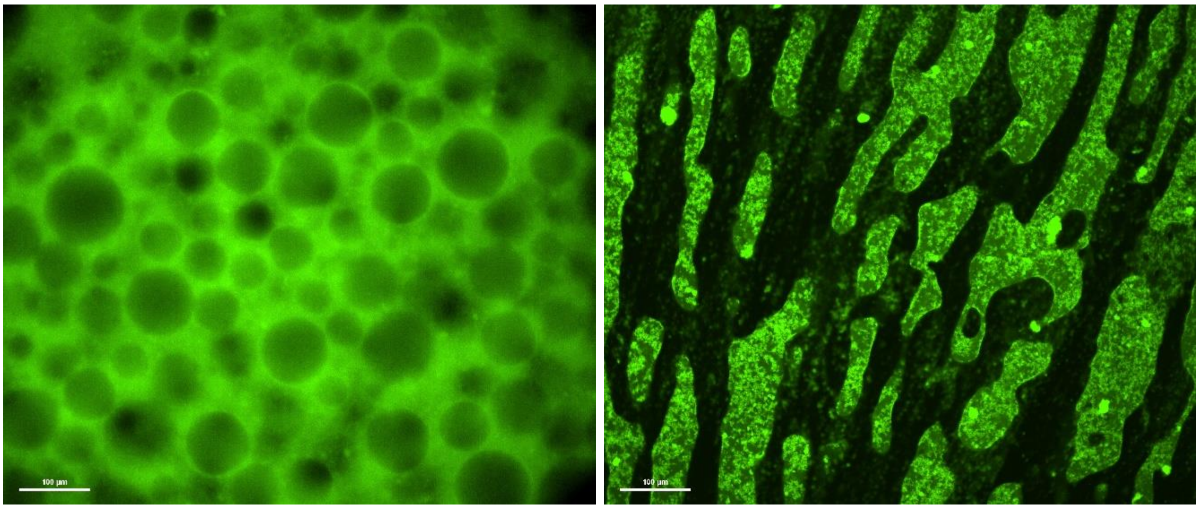

Figure S3. Fluorescence microscope images of the PEO 100 kDa/dextran emulsions (top phase) formed in the presence of 1 wt% silica and 0.090 wt% PDADMAC. **Left:** without; **right:** with pressing by cover slip. The horizontal bar denotes 100 µm.

.

Fluorescence microscope images of PEO100kDa/dextran emulsion systems at 4 wt % silica

The same two types of protocols as described previously for Figure S2 were applied during microscopic investigations. In each case, the emulsions were studied after one day of their synthesis.

Without squeezing with cover slip **Squeezing with coverslip**

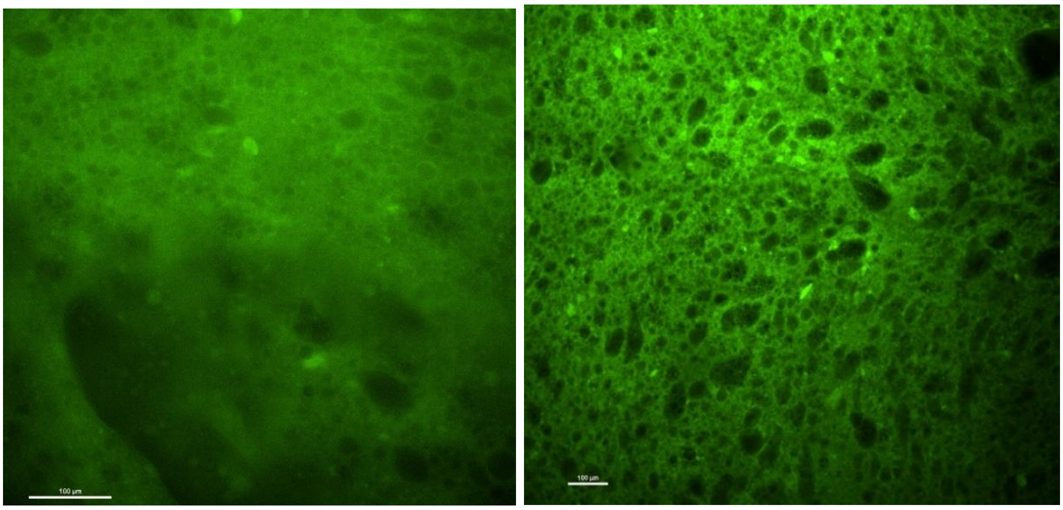

Figure S4. Fluorescence microscope images of the PEO 100 kDa/dextran emulsions (top phase) formed in the presence of 4 wt% silica and 0.36 wt% PDADMAC. **Left:** without; **right:** with pressing by cover slip. The horizontal bar denotes 100 μm.
